# Good corporate governance and corporate sustainability performance in Indonesia: A triple bottom line approach

**DOI:** 10.1016/j.heliyon.2021.e06453

**Published:** 2021-03-13

**Authors:** Bambang Tjahjadi, Noorlailie Soewarno, Febriani Mustikaningtiyas

**Affiliations:** Faculty of Economics and Business, Airlangga University, Indonesia

**Keywords:** Good corporate governance, Corporate sustainability performance, Triple bottom line, Agency theory, Sustainability theory, Upper echelon theory

## Abstract

This study aims to investigate the effect of good corporate governance (GCG) on corporate sustainability performance (CSP) using the Triple Bottom Line (TBL) approach in a two-tier GCG system. GCG is measured by the size and education background of board of commissioners (BoC) and top management team (TMT). CSP consists of economic, social, and environment sustainability performance. As many as 117 sample data were collected from the financial statements, annual reports and sustainability reports of non-financial companies listed on the Indonesia Stock Exchange (IDX) for the period of 2013–2017. Multiple regression analysis was employed to test the hypotheses studied with the following results. First, BoC education has a negative effect on economic and environmental sustainability performance and no effect on social sustainability performance. Second, BoC size has a positive effect on economic sustainability performance, a negative effect on social sustainability performance and no effect on environmental sustainability performance. Third, CEO's education has a negative effect on economic sustainability performance, and no effect on environmental and social sustainability performance. Fourth, TMT size has a negative effect on economic and environmental sustainability performance and no effect on social sustainability performance. Contributions, limitations and implications of the study are also discussed.

## 1 Introduction

Sustainability has become the concern of all nations. All United Nations (UN) member states adopted the Sustainable Development Goals (SDGs) in 2015 in order to end poverty, protect the planet and ensure prosperity by 2030. In the era of sustainable development, all companies are demanded by their stakeholders to increase awareness when carrying out their corporate responsibilities including dealing with global warming and human rights problems (Agnolucci and Arvanitopoulos, 2019; Alam et al., 2019; Shahbaz et al., 2020). Stakeholders expect that the company will sustainably realize its vision and mission. To realize its vision and mission, the company must build the stakeholder's trust. The Global Reporting Initiative (GRI, 1997) stated that trust must be maintained to achieve corporate sustainability.

CSP is the performance that is expected to continue in the long run by carrying out business activities that maintain the economic, social, and environmental welfare of society (Formentini and Taticchi, 2016; Hassini et al., 2012). Many international companies have used GRI as an indicator in their reporting (Fonseca et al., 2014; Hussain et al., 2018). The GRI guidelines have changed in recent years. There are six versions of GRI, namely GRI-G1 (2000), GRI-G2 (2002), GRI-G3 (2006), GRI G3.1 (2011), GRI G4 (2013) and GRI-Standards (2016).For the data availability, this study employs GRI-G4 which consists of 9 economic indicators, 34 environmental indicators and 48 social indicators (GRI, 2020) to assess CSP.

CSP highly depends on the quality of GCG because an effective GCG implementation will maintain the trust of stakeholders. There are two GCG systems adopted by countries in the world, namely the one-tier system and the two-tier system (Pellegrini et al., 2016). In a one-tier system, the board of directors (BoD) act as a supervisor and executor. In Indonesia which follows a two-tier system, the supervisory role is carried out by the BoC (*Dewan Komisaris*) while the BoD (*Dewan Direksi*) or TMT manages the company as the executor. The separation of roles between the BoC and the BoD in a two-tier system will improve the quality of the supervision and increase the transparency in decision making (Pellegrini et al., 2016). BoC are responsible for supervising and advising the BoD regarding the strategy and decision-making processes (OJK, 2007). A strong GCG implementation will reduce the agency problems in the company (Adams et al., 2015; Nadeem et al., 2017). The boards are fully responsible for sustainability performance because they determine the rules and decision making in the company (Díaz-Fernández et al., 2020; Krechovská and Procházková, 2014; Naciti, 2019).

GCG plays an important role in enhancing CSP. By implementing GCG, the stakeholder's trust in the company's sustainability performance will increase (Hussain et al., 2018). A strong GCG has five principles, namely fairness, accountability, responsibility, transparency and independence (Burak et al., 2017). The implementation of these principles forms the basis for reporting sustainability performance. Companies need to strengthen their GCG in order to reduce the agency problems arising from the conflict of interest between shareholders and agents (Naciti, 2019; Schäuble, 2019; Terjesen et al., 2014). The boards are the main parties who are in charge of strengthening GCG and maintaining the trust and interests of stakeholders by supervising and directing the managers so then they can make appropriate decisions (Naciti, 2019). Paniagua-Dominguez et al. (2018) and Zona et al. (2018) stated that the boards have a big impact on the company's performance.

To explain GCG-CSP relationship, this study employs three theories, namely agency theory (Jensen and Meckling, 1976), upper echelons theory (Hambrick and Mason, 1984) and sustainability theory (Meadows et al., 1972). Agency theory is used to explain the role of boards as the crucial part of GCG structure and mechanism. Agency theory states that shareholders as the principal and management agent have different interests (Chams and García-Blandón, 2019). GCG has a crucial role in overcoming the conflict between the principal and the agent. Regarding corporate sustainability, agency theory emphasizes that the board mechanism implementing social sustainability will provide benefits to the company (Chams and García-Blandón, 2019). Thus, based on agency theory, GCG will improve CSP. Upper echelon theory is employed in this study to explain that CSP is determined by the decisions of top leaders. Upper echelons theory focuses on the characteristics of top leaders assessed by several dimensions such as educational background and prior experience (Hambrick and Mason, 1984). Papadimitri et al. (2020) stated that education not only come from the things that have been learned but also from the intellectual ability of each individual. A higher level of education will increase the team's ability to find solutions to complex problems, therefore leaders' education background will affect the company's performance. Sustainability theory is used to explain that the leaders need to balance economic, social and environmental issues in order to achieve a better CSP. Sustainability theory states that society attempts to prioritize the social responses to environmental and economic problems. This social response is expected to meet the needs of the present and future generations (WCED, 1987). Krechovská (2013) stated that GCG affects sustainability performance including social, economic, and environmental welfare. The effect of GCG on sustainability will also increase the corporate value and CSR (Jaimes-Valdez and Jacobo-Hernandez, 2016; Klettner et al., 2014; Sharma and Khanna, 2014). Matten and Moon (2008) stated that corporate social responsibility (CSR) is an important instrument used to build sustainability performance. Činčalová and Prokop (2019) defines corporate social responsibility (CSR) as “an optional concept of socially responsible conduct beyond the legitimate commitments of the company that integrates the social, environmental and economic part and therefore it satisfies the objectives of all the interested parties”. Referring to the definition, corporate social performance is the performance of a firm that integrates the social, environmental and economic dimensions. In conclusion, those three theories are relevant to explain the relationship between GCG and CSP which becomes the focus of this study.

There is a research gap regarding the effect of GCG on CSP because the results of the previous studies are still inconsistent. This research gap justify the need for this study. In term of board size, Biswas et al. (2018) revealed that board size has a positive effect on social and environmental performance. Hussain et al. (2018) demonstrated that board size does not have significant effect on environmental, social and economic performance. In term of education, Ararat et al. (2015) and Yang et al. (2019) proved that the board's education has positive effect on corporate performance. Lindorff et al. (2013) found that there is no relationship between the CEO's MBA education, their business or other qualifications on the company's financial performance. Aggarwal et al. (2019) stated that the board demography (gender, age, education and tenure) has a negative effect on company performance. This study focuses on the four variables of GCG structure, namely BoC size, BoC education, TMT size, CEO's education, and three elements of CSP, namely economic, social, and environment sustainability performance.

The implementation of social and environmental responsibility in Indonesia is regulated by the Limited Liability Companies Law since the year of 2007. This law also regulates corporate governance practices, including the obligations of BoC and TMT. The implementation of GCG for go public companies in Indonesia is further regulated by some regulations issued by the Indonesian Financial Services Authority. The sustainability finance becomes the major issue in the Authority's master plan in the next five year. The objective of this study is to investigate the effect of GCG on CSP using a Triple Bottom Line (TBL) approach in Indonesia as a country that follows a two-tier GCG system. Specifically, this study has two problem statements: (1) Does GCG (BoC size and education) has a positive effect on CSP (economic, social, and environment)?; and (2) Does GCG (TMT size and CEO's education) has a positive effect on CSP?

This study continues the works of previous scholars, especially Hussain et al. (2018) with the following differences. First, as suggested by Hussain et al. (2018), this study uses other elements of GCG, namely education background of BoC and CEO. Second, this study is conducted in a two-tier GCG system adopted by companies in Indonesia. Third, it employs GRI-G4 to measure CSP. Finally, it focuses on non-financial companies listed on the IDX. This study is important for Indonesia because this country is one of the emerging markets, and it still has economic, social, and environmental issues such as corruption, gender, underage child employment and environmental issues (plastic waste, haze, river pollution, and forest degradation).

## 2 Literature review and hypothesis development

In agency theory, Jensen and Meckling (1976) stated that there is a conflict of interest between the principal (shareholders) and the agent (management). The agency problem emerges when the principal who has ownership of the company is separated from the agent who manages the company according to the interests of the principal (Poletti-Hughes and Briano-Turrent, 2019; Schillemans and Bjurstrøm, 2019; Teece, 2019). In reality, the shareholders do not know whether the agent has managed the company on behalf of their interests or not (Ang and Cheng, 2016; Buxton and Rivers, 2014). The emergence of agency problems requires GCG. In this case, the role of BoD is very crucial when seeking to protect the shareholders’ interests (Boubaker et al., 2016; Chari et al., 2019; Schillemans and Bjurstrøm, 2019; Vitolla et al., 2020). The presence of BoD will maximize firm value and reduce the agency costs so then the company performance will also become better (Chari et al., 2019; Teece, 2019; Vitolla et al., 2020). Thus, agency theory underlies the importance of BoC and TMT in implementing GCG to increase CSP.

In sustainability theory, Meadows et al. (1972) explained that companies must respond to society's priorities, namely their social, environmental and economic welfare. This response has to meet the needs of the present and future generations (WCED, 1987). The concept of sustainability is currently growing and applied in the context of corporate sustainability. Artiach et al. (2010) and Pemer et al. (2020) both stated that businesses and investment will improve through balancing the needs of current and future stakeholders. As proposed by Elkington and Rowlands (1999), corporate sustainability is operationalized through the concept of the triple bottom line (TBL) consisting of economic, social and environmental factors. Markley and Davis (2007) and Pemer et al. (2020) have also proved that companies focusing on TBL have increased their competitive advantage. Thus, sustainability theory underlies the crucial roles of BoC and TMT in implementing GCG that can balance economic, social and environmental activities to achieve CSP.

In upper echelons theory, Hambrick and Mason (1984) explained that company performance is judged by the decision making of the company's top executives. Several of the previous studies have used the characteristics of the top executives such as age, ethnicity, experience, education and functional background as proxies to be observed (Pemer et al., 2020; Petrovsky et al., 2015). Proxies in the form of individual psychology form the perspective of the top executives when interpreting social, economic and environmental problems and responding to how top executives deal with these problems (Petrovsky et al., 2015; Strand, 2014). This theory begins to consider the importance of the psychological condition of the top management team and influences decision making (Hambrick, 2007). This theory is used to explain the company's response to the problem of sustainability because the issue has emerged and is growing rapidly (Connelly et al., 2011). The response to the sustainability issue depends on the background and experience of the top executives and top management team in terms of managing information. This response can be in the form of innovation, strategy change and the creation of a corporate social strategy (Kanashiro and Rivera, 2019; Mazutis, 2013; Qian et al., 2013). As stated by Hambrick (2007) and Khan et al. (2019), the more experienced the top executives and top management team are, the more proper the decision making to do with complex problems. Upper echelon theory underlies the role of the leaders' characteristics such as their education in a successful GCG implementation to increase CSP.

### 2.1 Board size and CSP

Agency theory, sustainability theory and upper echelon theory explain that BoC has an important role in implementing GCG effectively to achieve CSP. The effectiveness of the board is reflected by the size of the board. As explained by Berraies and Rejeb (2019) and Saidat et al. (2019), the big size of the board will bring in many advantages. The company will have many different views and ideas which will create a better strategy. Hussain et al. (2018) revealed that the smaller the board size, the more of a workload there is for each board member. This will reduce the quality of their supervision. The studies by Arena et al. (2014) and Said et al. (2009) demonstrated that BoD size has a positive effect on environmental disclosure. Husted and de Sousa-Filho (2019) proved that board size has a positive effect on economic, social and governance disclosure (ESG). Chams and García-Blandón (2019) also revealed that board size has a positive effect on sustainable performance. Some of the previous scholars have also supported the statement that board size has a positive effect on CSR disclosure (Esa and Ghazali, 2012; Jizi et al., 2014; Majeed et al., 2015). In conclusion, theories and previous studies support that the BoC size matters in an effective GCG implementation to increase CSP. Considering the previous arguments, the two-tier system adopted by companies in Indonesia and the TBL approach, the following hypotheses are proposed:

H1 *BoC size has a positive effect on economic sustainability performance*

H2 *BoC size has a positive effect on environmental sustainability performance*

H3 *BoC size has a* positive *effect on social sustainability performance,*

### 2.2 President of BoC's education and CSP

Referring to agency theory and sustainability theory, the TBL approach encourages company leaders to implement their vision to survive in the long term (Meadows et al., 1972). Upper echelons theory states that the background of the company leaders influences the company strategy and performance (Hambrick and Mason, 1984). As revealed by Chang et al. (2015), Harjoto et al. (2014) and Oh et al. (2019), the diversity of the different educational backgrounds improves the quality of the resources so then they are able to address the various stakeholders’ interests and increase the social responsibility performance more effectively.

The study by Jhunjhunwala and Mishra (2012) demonstrated that the diversity in terms of the educational background also increases the technological progress made in the company. The studies by Kagzi and Guha (2018), Shahrier et al. (2020) and Yang et al. (2019) support the statement that the education of the board has a positive effect on the company's performance. Colakoglu et al. (2020), Ma (2019) and Mascarenhas et al. (2020) explained that councils with a higher level of education and experience abroad have a positive effect on CSR performance. Martín and Herrero (2020) also stated that the BoD's education has a significant positive effect on sustainable environmental performance. Hassan and Marimuthu (2018) and Pereira and Filipe (2018) also proved that educational background has a positive effect on the company's financial performance. Considering the previous arguments, the two-tier system adopted by the companies in Indonesia, and the TBL approach, the following hypotheses are proposed:

H4 *President of BoC's education has a positive effect on economic sustainability performance*

H5 *President of BoC's education has a positive effect on environmental sustainability performance*

H6 *President of the BoC's education has a positive effect on social sustainability performance*

### 2.3 TMT size and CSP

In line with sustainability theory, the adoption of the GRI G4 standard will guide the company in identifying the weaknesses and improve sustainability performance. TMT size is a major consideration in agency theory because it affects firm performance. The previous studies have not examined the issue of TMT size as much. In Indonesia, it refers to the top executives who have a direct influence when it comes to determining the company's strategy. Several of the previous studies have found there to be a relationship between the TMT and decision making. This decision making is influenced by the scores, beliefs, views and judgment of the top managers (Díaz-Fernández et al., 2020). Managerial decisions are made based on the presence of complex, uncertain and ambiguous information. The bigger the size of the TMT, the slower the team's communication speed. This leads to potential information asymmetry (Jaw and Lin (2009); Kearney and Gebert, 2009). Arena et al. (2019) proved that TMT size has a significant negative effect on the company's financial performance. Lai and Liu (2018) also showed that a greater TMT size has a negative effect on the company's investment efficiency. Based on the previous arguments, the following hypotheses are proposed:

H7 *TMT size has a negative effect on economic sustainability performance*

H8 *TMT size has a negative effect on environmental sustainability performance*

H9 *TMT size has a negative effect on social sustainability performance*

### 2.4 CEO's education and CSP

In line with sustainability theory, the company leaders who adopt the TBL approach to assess sustainability performance will increase their competitive advantage and survive in the long-term (Meadows et al., 1972). The upper echelons theory also states that the background of the company leaders will affect how to view the internal and external problems and make decisions (Hambrick and Mason, 1984). Saidu (2019) and Wu et al. (2011) described that the leaders who have a good education and strong experience will improve the high managerial skills present, so they will guarantee the company's sustainability. Frydman (2019) and King et al. (2016) stated that having a background in business education can improve the managerial skill rather than technical skill. Hsu et al. (2013) and Papadimitri et al. (2020) found that CEOs with an MBA background have stronger strategies.

A CEO with the higher level of education increases the prospect of the company's sustainability due to the increased capability to manage the company (Abernethy et al., 2019; Javakhadze et al., 2016; Kaur and Singh, 2019). Kokeno and Muturi (2016), Naseem et al. (2019) and Saidu (2019) found that the CEO's education has a positive effect on the company's financial performance. Lewis et al. (2014) and Tran and Pham (2020) also revealed that the CEO's education level has a positive effect on corporate environmental performance. Moreover, Manner (2010) supported the statement that the CEO's educational background improves the company's corporate social performance. Finally, Shahab et al. (2020) proved that the CEO's education has a positive effect on sustainable performance. Based on the previous arguments, the two-tier system adopted by the companies in Indonesia and the TBL approach, the following hypotheses are proposed:

H10 *The CEO's education has a positive effect on economic sustainability performance*

H11 *The CEO's education has a positive effect on environmental sustainability performance*

H12 *The CEO's education has a positive effect on social sustainability performance*

**Figure 1:**
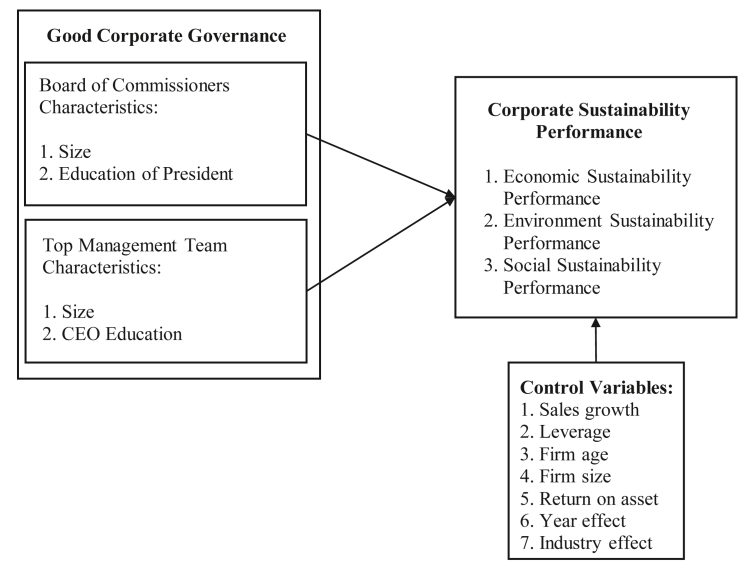
Research framework.

Figure 1 shows the research framework based on the literature review and hypothesis development. This framework depicts how GCG proxied by the characteristics of the Board of Commissioners (size and education) and the Top Management Team (size and education) affect the three dimensions of corporate sustainability performance (economic, environment, social) and the control variables (sales growth, leverage, firm age, firm size, return on asset, year effect and industry effect).

## 3 Methods

### 3.1 Data source

The secondary data was collected from the ORBIS database in addition to the annual reports and sustainability reports of the non-financial companies. The company's annual report was obtained from the official IDX website and the company's official website. The sustainability reports were also obtained from the company's official website for the period of 2013–2017. The measurement of sustainability performance was adopted from the GRI-G4 guidelines which were accessed from the official GRI website, www.globalreporting.org specifically.

### 3.2 Sample

This study employed the purposive sampling method. The sampled company must meet the following criteria: (1) non-financial company listed on the IDX for the period of 2013–2017; (2) having annual reports with complete information for the period of 2013–2017; (3) having sustainability reports or statements for the period 2013–2017; and (4) employing the GRI-G4 guidelines for their sustainability reports and sustainability statements in the annual reports for the period of 2013–2017. Table 1 shows the sample selection of this study.

**Table 1:** Sample selection.

Criteria	Year					TOTAL FIRM YEAR
	2013	2014	2015	2016	2017	
Non-financial Companies listed in the IDX with complete information	407	405	407	409	416	2044
Non-financial companies which do not use GRI guidelines	-381	-373	-377	-380	-398	-1909
**Non-financial companies that uses GRI Guidelines**	**26**	**32**	**30**	**29**	**18**	**135**
Non-financial companies which do not use GRI-G4	-7	-2	0	-1	-8	-18
**Non-financial companies which use GRI- G4 Guidelines**	**19**	**30**	**30**	**28**	**10**	**117**

Source: ORBIS database, IDX website, and the companies' websites.

Source: ORBIS database, IDX website, and the companies' websites.

Table 2 shows the sampled companies based on the business sector as defined by the ORBIS database. There are 6 business sectors among the 12 business sectors covered in this study.

**Table 2:** Business sectors of the sampled companies.

SIC Codes	Sector	Sample data
0100–0999	Agriculture, Forestry, and Fishing	12
1000–1499	Mining	39
1500–1799	Construction	4
2000–3999	Manufacturing	30
4000–4999	Transportation, Communication, Electric, Gas and Sanitary service	20
5000–5199	Wholesale Trade	12
**TOTAL SAMPLE DATA**		**117**

Multicollinearity, heteroscedasticity, and autocorrelation tests show the following results. First, each independent variable has a VIF that is less than 10, meaning that the research models do not have multicollinearity problems. Second, the Breusch-Pagan and Wald tests show that there are no heteroscedasticity problems in all models. The Wooldridge test shows that the residuals generated from all models do not have autocorrelation issue.

### 3.3 Empirical model

This study has investigated the effect of GCG (proxied by the characteristics of the BoC and TMT) on corporate sustainability performance. Employing multiple linear regression analysis, the models were formulated as follows.

Model 1: Effect of the characteristics of the BoC and TMT on economic sustainability performance

*EcSP*_*it*_ = *α* + *β*_*1*_*ComSize*_*it*_ + *β*_*2*_*PComEd*_*it*_ + *β*_*3*_*DirSize*_*it*_ + *β*_*4*_*PDirEd*_*it*_ + *γControl*_*it*_ + *е*

Model 2: Effect of the characteristics of the BoC and TMT on environmental sustainability performance

*EnvSP*_*it*_ = *α* + *β*_*1*_*ComSize*_*it*_ + *β*_*2*_*PComEd*_*it*_ + *β*_*3*_*DirSize*_*it*_ + *β*_*4*_*PDirEd*_*it*_ + *γControl*_*it*_ + *е*

Model 3: Effect of the characteristics of the BoC and TMT on social sustainability performance

*SocSP*_*it*_ = *α* + *β*_*1*_*ComSize*_*it*_ + *β*_*2*_*PComEd*_*i*t_+ *β*_*3*_*DirSize*_*it*_ + *β*_*4*_*PDirEd*_*it*_+ *γControl*_*it*_ + *е*

Notes:

α: Constant
β_1_ComSize_it_: BoC size
β_2_PComEd_it_: President of the BoC's education
β_3_DirSize_it_: TMT size
β_4_PDirEdit: CEO's education
γControl_it_: Control variables
е: Error term

### 3.4 Definition and measurement

#### 3.4.1 BoC size

BoC size is defined as the total number of commissioners in the company. Referring to the work of Chams and García-Blandón (2019), Chong et al. (2018) and Hussain et al. (2018), the following formula was adopted to measure the size of the BoC.

BoardofCommissioners(BoC)size=∑thenumberofallcommissionersinthecompany

#### 3.4.2 President of BoC’ education

The president of the BoC's education is defined as the education background of the president of the BoC. To measure it, this study employed education background scoring. The president of the BoC who has a Bachelor's, Master's, MBA and PhD educational background is assigned a dummy variable with a value of 1, otherwise they are assigned the value of 0. The following scoring formula was adopted from the work of Papadimitri et al. (2020).

Scoring=Bachelor+2×Master+2×MBA+3xPhD

#### 3.4.3 TMT size

TMT size is defined as the total number of top executives (*dewan direks*i) in the company. Referring to the work of Hussain et al. (2018), Zahid et al. (2020) and Zaid et al. (2020), the following formula was employed to measure the size of the TMT.

TMTsize=∑thenumberoftopexecutivesinthecompany

#### 3.4.4 CEO's education

The CEO's education is defined as the level of education background that has been taken by the CEO. It is accessed using the education background scoring. CEO with a Bachelor's, Master's, MBA and PhD educational background were assigned the value of 1, otherwise they were assigned the value of 0. The following scoring formula was adopted from Papadimitri et al. (2020).

Scoring=Bachelor+2×Master+2×MBA+3xPhD

#### 3.4.5 Corporate sustainability performance

Corporate sustainability performance is defined as the disclosure of sustainability indicators in the annual and sustainability report consisting of economic, environmental, and social performance indicators. These indicators are based on the GRI-G4 indicators with a total of 91 items. These indicators consist of 9 items for the economic indicators, 34 items for the environmental indicators, and 48 items for the social indicators. The corporate sustainability disclosure index was used to measure sustainability performance. This measure assigns the value of 1 for each disclosed item in the sustainability report and the sustainability statement and 0 if the item is not disclosed. This measurement was adopted from the work of Haniffa and Cooke (2005) and Zaid et al.(2020).

$$CSDIj=\;\frac{\Sigma Xij}{nj}$$

Note:

CSDIj: Corporate sustainability disclosure index for j company
nj: total items on j company, nj ≤ 91
Xij: Total items for sustainability disclosure (1 if disclosed and 0 if not disclosed). So, 0 ≤ CSDIj ≤ 1

#### 3.4.6 Firm size

The firm size is measured by the following formula:

Firm size = Ln Total assets

## 4 Results and discussions

### 4.1 Descriptive statistics

Table 3 shows the descriptive statistics of the study. It shows the mean, standard deviation and minimum and maximum values of each variable studied. Based on the mean and standard deviation values, the data diversity of the main variables tends to be small.

**Table 3:** Descriptive statistics.

Variabel	Mean	St.Dev	Minimum	Maximum
*Economic Sustainability Performance*	0.502	0.235	0.000	1.000
*Environment Sustainability Performance*	0.338	0.218	0.000	0.912
*Social Sustainability Performance*	0.334	0.197	0.063	0.875
President of BoC's education	2.889	1.865	0.000	6.000
BoC Size	6.111	1.804	3.000	12.000
CEO's Education	2.376	1.337	0.000	8.000
TMT Size	6.162	1.814	3.000	11.000
Sales Growth	0.019	0.185	-0.423	0.708
Leverage	0.558	0.294	0.133	1.898
Firm Age	47	26	13	156
Firm Size	23.790	1.114	21.096	26.291
*ROA*	0.063	0.118	-0.644	0.421

### 4.2 Pearson correlation

Table 4 presents the results of the Pearson correlation. The results show that there is a significant positive correlation between three sustainability performance dimensions (correlation coefficient between economic SP and environmental SP: 26.5% with p-value<0.01); between economic SP and social SP: 45.8 % with p-value<0.01) and between social SP and environmental SP: 58.8% with p-value<0.01). The positive sign of the coefficient means that the three sustainability performance measures are moving in the same direction.

**Table 4:** Pearson correlation.

	EcSP	EnvSP	SocSP	PndKut	UDKom	PnDut	UDDir	SG	Lev	FA	FS	ROA
EcSP	1.000											
EnvSP	0.265^∗∗∗^	1.000										
	(0.004)											
SocSP	0.458^∗∗∗^	0.586^∗∗∗^	1.000									
	(0.000)	(0.000)										
PndKut	0.026	0.060	0.113	1.000								
	(0.783)	(0.523)	(0.223)									
UDKom	-0.063	-0.035	-0.102	0.101	1.000							
	(0.502)	(0.707)	(0.276)	(0.278)								
PnDut	0.058	0.089	-0.005	0.093	0.354^∗∗∗^	1.000						
	(0.534)	(0.340)	(0.954)	(0.319)	(0.000)							
UDDir	-0.007	0.087	-0.004	-0.053	0.237^∗∗^	0.106	1.000					
	(0.944)	(0.351)	(0.966)	(0.569)	(0.010)	(0.255)						
SG	-0.011	-0.074	-0.038	0.014	0.031	-0.070	0.058	1.000				
	(0.903)	(0.431)	(0.684)	(0.882)	(0.741)	(0.453)	(0.537)					
Lev	0.008	-0.194^∗∗^	-0.023	-0.202^∗∗^	-0.284^∗∗∗^	-0.202^∗∗^	-0.186^∗∗^	-0.300^∗∗∗^	1.000			
	(0.930)	(0.036)	(0.809)	(0.029)	(0.002)	(0.029)	(0.045)	(0.001)				
FA	0.066	0.027	0.043	0.084	0.031	0.200^∗∗^	0.067	-0.113	0.154^∗^	1.000		
	(0.480)	(0.772)	(0.641)	(0.366)	(0.741)	(0.031)	(0.476)	(0.225)	(0.097)			
FS	0.123	0.002	0.121	0.048	0.470^∗∗∗^	0.152	0.394^∗∗∗^	-0.094	0.023	0.036	1.000	
	(0.187)	(0.982)	(0.194)	(0.604)	(0.000)	(0.102)	(0.000)	(0.313)	(0.808)	(0.697)		
ROA	-0.051	-0.014	0.059	0.151	0.076	0.055	0.276^∗∗∗^	0.288^∗∗∗^	-0.411^∗∗∗^	0.159^∗^	-0.134	1.000
	(0.587)	(0.881)	(0.524)	(0.103)	(0.418)	(0.555)	(0.003)	(0.002)	(0.000)	(0.087)	(0.149)	

Note: ∗*p* < 0.1, ∗∗*p* < 0.05, ∗∗∗*p* < 0.01.

### 4.3 Hypothesis testing

#### 4.3.1 Model 1 (economic sustainability performance)

Table 5 presents the results of the hypothesis testing regarding the effect of the characteristics of the BoC on economic sustainability performance. H1 states that the BoC size has a positive effect on economic sustainability performance. The results show that BoC size has a significant positive effect on economic sustainability performance (coefficient: 0.05316; p-value<0.1). H1 is thus supported. H4 states that the president of the BoC's education has a positive effect on economic sustainability performance. The results show that the president of the BoC's education has a negative effect on economic sustainability performance (coefficient: -0.03974; p-value<0.05). Although the results have a statistically significant effect, the direction of the effect is not as expected. H4 is not supported.

**Table 5:** Results of model 1: Economic sustainability performance.

Variable	Exp. Sign	Coefficient	P>|t|
President of BoC's education	+	-0.03974	0.042∗∗
Board of Commissioners size	+	0.05316	0.056∗
CEO's Education	+	-0.05128	0.021∗∗
TMT size	-	-0.06470	0.001∗∗∗
Sales Growth		0.45550	0.002∗∗∗
Leverage		0.34945	0.015∗∗
Firm age		0.00069	0.367
Firm Size		-0.42187	0.001∗∗∗
Profitability(ROA)		-0.00075	0.996
Year			
2014		-0.01292	0.752
2015		0.03077	0.554
2016		0.03843	0.414
2017		-0.05111	0.564
Cons		10.60383	0.001∗∗∗
R Square		0.4058	

Note:∗ p-value<0.1, ∗∗ p-value<0.05, ∗∗∗ p-value<0.01.

Table 5 also shows the effect of the characteristics of the TMT on economic sustainability performance. H7 states that TMT size has a negative effect on economic sustainability performance. The results show that TMT size has a significant negative effect on economic sustainability performance (coefficient: -0.0647; p-value<0.01). H7 is thus supported. H10 states that the CEO's education has a positive effect on economic sustainability performance. The results show that the CEO's education has a significant negative effect on economic sustainability performance (coefficient: -0.05128; p-value<0.05). Although this proves that the CEO's education has an effect on economic sustainability performance, the direction of the effect is not as expected. Therefore H10 is not supported.

#### 4.3.2 Model 2 (environmental sustainability performance)

Table 6 shows the results of the effect of the characteristics of the BoC and CEO on environmental sustainability performance. H2 states that the BoC size has a positive effect on environmental sustainability performance. However, the empirical results demonstrate that BoC size has no effect on environmental sustainability performance. H2 is thus not supported. H5 states that the president of the BoC's education has a positive effect on environmental sustainability performance. The empirical results reveal that the president of the BoC's education has a negative effect on environmental sustainability performance (coefficient: -0.02939; p-value<0.01). Although the result shows a statistically significant effect, the direction of the effect is not as expected. H5 is thus not supported.

**Table 6:** Results of model 2: Environmental sustainability performance.

Variable	Exp Sign	Coefficient	P>|t|
President of BoC's education	+	-0.02939	0.009∗∗∗
Board of Commissioners size	+	0.00885	0.605
CEO's Education	+	-0.01145	0.349
TMT size	-	-0.02443	0.006∗∗∗
Sales Growth		0.18371	0.040∗∗
Leverage		-0.03839	0.724
Firm age		0.00137	0.016∗∗
Firm Size		-0.28608	0.011∗∗
Profitability(ROA)		0.31563	0.006∗∗∗
Year			
2014		0.01820	0.676
2015		0.06027	0.205
2016		0.02005	0.687
2017		-0.97894	0.101
Constant		7.269186	0.007∗∗∗
R Square		0.3285	

Note: ∗ p-value<0.1, ∗∗ p-value<0.05, ∗∗∗ p-value<0.01.

Table 6 also reveals the effect of the characteristics of the TMT on environment sustainability performance. H8 states that the TMT size has a negative effect on environmental sustainability performance. The results prove that the TMT size has a significant negative effect on economic sustainability performance (coefficient: -0.02443; p-value<0.01). In conclusion, H8 is supported. H11 states that the CEO's education has a positive effect on environment sustainability performance. The results reveal that the CEO's education has no effect on environmental sustainability performance. Therefore H11 is not supported.

#### 4.3.3 Model 3 (social sustainability performance)

Table 7 presents the results of the characteristics of the BoC and TMT on social sustainability performance. It shows that the BoC size has a significant negative effect on social sustainability performance (coefficient: -0.03074; p-value<0.10). While the results show a statistically significant effect, the direction of the effect is not as expected. Therefore H3 is not supported. Table 7 also reveals that the president of the BOC's education, the CEO's education and TMT size do not have a significant effect on social sustainability performance. H5, H9 and H12 are thus not supported.

**Table 7:** Results of model 3: Social sustainability performance.

Variable	Exp. Sign	Coefficient	P>|z|
President of BoC's education	+	0.00083	0.950
Board of Commissioners size	+	-0.03074	0.058∗
CEO's Education	+	-0.01575	0.306
TMT size	-	-0.01732	0.216
Sales Growth		-0.07746	0.524
Leverage		-0.06105	0.674
Firm age		0.00036	0.667
Firm Size		0.06395	0.036∗∗
Profitability(ROA)		0.42623	0.138
Year			
2014		-0.02927	0.385
2015		-0.03940	0.493
2016		-0.06603	0.137
2017		-0.06465	0.232
Constant		-0.74438	0.252
R Square		0.1603	

Note: ∗ p-value<0.1, ∗∗ p-value<0.05, ∗∗∗ p-value<0.01.

## 5 Discussion

Table 8 presents the summary of hypotheses testing. The empirical results reveals that not all hypotheses based on agency theory, sustainability theory, and upper echelon theory are supported in the research setting of the IDX. The results can be classified into two categories: (1) hypotheses are supported (significant-same sign as expected); (2) hypotheses are not supported (significant-different sign or not significant. The following sections present the detail discussion on each hypothesis.

**Table 8:** Summary of the hypothesis testing.

Hypothesis	Expected Sign	Resulted Sign	Statistically Significance	Description	Decision
H1: BoC size > ESP	+	+	S∗	Sig, same sign	Supported
H2: BoC size > EnSP	+	+	NS	Not sig, same sign	Not supported
H3: BoC size > SSP	+	-	S∗	Sig, different sign	Not supported
H4: President of BoC's education > ESP	+	-	S∗∗	Sig, different sign	Not supported
H5: President of BoC's education > EnSP	+	-	S∗∗∗	Sig, different sign	Not supported
H6: President of BoC's education > SSP	+	+	NS	Not sig, same sign	Not Supported
H7: TMT size > ESP	-	-	S∗∗∗	Sig, same sign	Supported
H8: TMT size > EnSP	-	-	S∗∗∗	Sig, same sign	Supported
H9: TMT size > SSP	-	-	NS	Not sig, same sign	Not Supported
H10: CEO's Education > ESP	+	-	S∗∗	Sig, different sign	Not Supported
H10: CEO's Education > EnSP	+	-	NS	Not sig, different sign	Not Supported
H10: CEO's Education > SSP	+	-	NS	Not sig, different sign	Not Supported

Note: ∗ p-value<0.1, ∗∗ p-value<0.05, ∗∗∗ p-value<0.01.

### 5.1 The effect of the BoC size on economic, environmental and social sustainability performance

The results of this study indicate that the BoC size has a significant positive effect on economic sustainability performance. This empirical result is theoretically expected and it is in line with the study by Chams and García-Blandón (2019) on multinational companies across different continents stating that board size has a positive effect on sustainable performance. Furthermore, Chams and García-Blandón (2019) also stated that the more board members there are, the better their performance in terms of paying attention to the stakeholders' interest. The study by Husted and de Sousa-Filho (2019) in Latin America also revealed that board size has a significant positive effect on ESG disclosure. Moreover, they also stated that the larger the board size, the greater the outlook for decision making. By assessing the top 25 board members and the bottom 25 board members in the Business Week ranking, Saidat et al. (2019) demonstrated that the more members of board there are, the higher the board's performance. In Indonesia, the results of this study support the previous studies in the one-tier system. A bigger BoC represents the stockholders' interest more efficiently and effectively in terms of receiving information and making better decisions, in addition to enhancing economic and financial performance. This also indicates that the economic performance is still the major focus of the board as the representative of the stockholders.

This study reveals that the BoC size has no significant effect on environmental sustainability performance. Although this finding do not match theoretical expectation, this is in line with the study conducted by Hussain et al. (2018) stating that there is no significant effect of board size on environmental sustainability performance. Ienciu et al. (2012) conducted a study on oil companies in Romania proving that the board size has no effect on environmental reporting in addition to stating that board size is not an effective measure when assessing environmental performance. Another study by Jo and Harjoto (2011) in the United States found that there is no significant effect due to board size on CSR performance because board size does not provide any benefit for the company. In Indonesia, the fact that BoC size has no effect on environmental sustainability performance suggests that the environmental issue is not the priority of the board. The big or the small size of the BoC will not affect environmental sustainability performance when the environmental issue does not provide any benefit for the company, therefore it is not the priority.

This study also indicates that the BoC size has a significant negative effect on social sustainability performance. This is different from the theoretical expectation, but this is in line with a study conducted in the US by Bai (2013) proving that board size has a significant negative effect on corporate social performance. The bigger the board size, the less effective the supervision of the company performance. Another study in the US by Hafsi and Turgut (2013) demonstrated that board size has a significant negative effect on social performance as a smaller board size will increase the innovation present in the corporate strategic decision making. Moreover, the study by Kassinis and Vafeas (2002) in the US revealed that board size has a significant negative effect on social performance for the reason that the more board members there are, the more inputs they get. This makes the decision making process more difficult. In Indonesia, a bigger BoC size has a negative effect on social sustainability performance which might be caused by the slow decision making process and lack of innovation.

### 5.2 The effect of the BoC president's education on the economic, environmental and social sustainability performance

The results of this study show that the president of the BoC's education has a significant negative effect on economic sustainability performance. This is in line with the study by Hasan et al. (2019) in Bangladesh revealing that board education has a significant negative effect on financial performance. Wellalage and Locke (2013) conducted a study in Sri Lanka and the results prove that board education has a negative effect on company performance. The board members with a higher education will reduce the level of performance when they do not understand the current conditions as they make biased decisions. The study by Mahadeo et al. (2012) in Mauritius revealed that the more diverse the board's education, the lower the company's performance. The board members with a specific educational background tend to focus only on their expertise and might sacrifice other aspects which are more important to the company's sustainability.

In Indonesia, the president of the BoC's education has a negative effect on economic sustainability performance. The following are some of the possible reasons. First, the education of the president that does not align with the company's business field might cause some potential problems in terms of economic performance. Second, an education level that does not match with that of the other members might also raise potential problems concerning economic performance. Third, a president with a high education background has more power to influence what financial information to disclose which can potentially reduce the quality of the economic disclosure.

This study has revealed that the president of the BoC's education has a significant negative effect on environmental sustainability performance. It also demonstrates that the president of the BoC's education has no significant effect on social sustainability performance. Schwartz and Bardi (2001) stated that culture shapes an individual's personality when viewing problems, including environmental and social issues. The cultural factors of the BoC and the lack of experience in the field of environment might determine how the BoC perceives environmental and social issues, not education.

The result is in line with the study by Chams and García-Blandón (2019) in the United States. This also supports the study by Post et al. (2011) in the setting of the European Union proving that there is no significant effect due to the board education background on corporate social responsibility (CSR) performance. The most dominant determinant of CSR is culture. Even though the education of the board members is high, each member has a different culture and values when looking at environmental issues. Schwartz and Bardi (2001) added that culture shapes a person's personality when viewing a problem. Social issues may not be the main concern of the BoC in Indonesia and therefore those issues are not related to their characteristics, including their education. This result also supports the study by Katmon et al. (2019) in Malaysia stating that the board members with a financial education background reduce the company's CSR disclosure due to the lack of experience in other fields. This therefore cannot clearly describe environmental sustainability performance and social sustainability performance.

### 5.3 Effect of TMT size on economic, environmental and social sustainability performance

The results of this study state that TMT size has a negative significant effect on economic and environmental sustainability performance. This finding is theoretically predicted and it is in line with with the study by Arena et al. (2019) in Italy which explains that TMT size has a negative effect on the company's financial performance. The bigger the TMT size, the more complex the communication process. Thus strategy and innovation become less efficient. A study by Lai and Liu (2018) in Taiwan proved that the big size of the TMT has a negative effect on the company's investment efficiency because the interaction between the members is inefficient and biased. Similar reasons might be applied to Indonesia, proving that the bigger the size of the TMT, the more complicated the communication and interaction process. The information provided to investors tends to be biased.

The results of this study prove that TMT size has no significant effect on social sustainability performance. This is in line with the results of the studies conducted in the UK and Italy (Aghion and Tirole, 1997; Rovelli, 2020) revealing that TMT size has no significant effect on meetings and decision making, including social sustainability decision making. In Indonesia, TMT size has no significant effect on social sustainability performance simply because social issues are not the main concern of the TMT.

### 5.4 The effect of CEO's education on economic, environmental and social sustainability performance

This study proves that the CEO's education has a significant negative effect on economic sustainability performance. This result is in line with the study by Datta and Guthrie (1994) that derived its sample from the Business Week, proving that the CEO's education has a negative significant effect on the company's financial performance. This happens because the CEO's education is not always in line with the finances of the company (Datta and Guthrie, 1994; Hambrick and Mason, 1984). Van Ness et al. (2010) conducted a study in the United States and stated that the CEO's education has a negative significant effect on financial performance. The study by Kaur and Singh (2019) in India also proved that CEOs with high intellectual abilities reduce the companies' financial performance due to their overconfidence.

This study proves that the CEO's education has no significant effect on the environmental and social sustainability performance. This is also in line with the study by Bhagat et al. (2010) in the United States revealing that the CEO's education has no effect on company performance due to their lack of business experience. The study by Gottesman and Morey (2006) in the United States revealed that the CEO's education background has no effect on the quality of the company's performance because it is not important anymore. CEOs have already achieved a high level of success in their life so the CEO's education is not an important factor when assessing the company's performance. In Indonesia, similar reasons might be applied.

## 6 Conclusions

This study aims to investigate the effect of good corporate governance (proxied by the characteristics of the BoC and TMT) on CSP using the TBL approach. Deriving the sample data from the non-financial companies listed on the IDX (Indonesia Stock Exchange) for the 2013–2017 period and by employing the GRI-G4 guidelines, the study concludes the following. First, not all hypotheses are supported. This implies that further studies are still needed. Second, the findings are beneficial for some policy recommendations. The Indonesian Financial Services Authority as one of regulators needs to consider the importance of the size and education of the BoC and TMT in formulating regulations to improve GCG and CSP. Investors, especially institutional investors, will benefit from considering the size and education of the BoC and TMT in their investment decisions. The findings also imply that the leaders need to maximize their role in improving the company's sustainability performance via GCG. The issues of GCG and sustainability are relatively new in Indonesia. Therefore, this study provides opportunities for various parties to improve GCG and corporate sustainability.

## 7 Contributions

From the theoretical perspective, this study provides the following contributions. First, it provides an empirical evidence for further development of agency theory, upper echelon theory and sustainability theory on a two-tier GCG system in Indonesia as an emerging market. Second, as the first study that employs the TBL approach in Indonesia, it provides a deeper insight regarding the phenomena of size and education of BoC and TMT in the IDX. Third, it is useful as a course mateial for students who take GCG course. Finally, by employing the GRI-G4 guidelines, it provides a new data set which is valuable for future studies.

From the practical perspective, this study provides useful information for various decision makers. First, the capital market regulator (Financial Service Authority) in Indonesia will get a better understanding when they make policies regarding the roles of BoC size, BoC education, TMT size, and CEO's education on each elements of CSP. Second, it is beneficial for potential investors who will assess the companies's performance and the characteristics of GCG as its determinants. Third, the management of companies will have a better understanding on the crucial roles of GCG characteristics in improving corporate sustainability. Finally, the society will improve their welfare from the increasing quality of GCG as well as increasing economic, social, and environment sustainability performance.

## 8 Limitations and future research

This study has the following limitations. First, it limits the sample size only non-financial companies listed in the IDX. Further studies are encouraged to investigate companies in the financial sector. Second, for data availability, this study limits the research period of 2013–2017 to conform with the GRI G4 guidelines. Future research needs to consider a longer period of study and employs other measurements. Finally, this study only considers the BoC size, president of the BOC's education, CEO's education and TMT size as the proxies of good corporate governance. The next researchers should consider other proxies of good corporate governance such as the boards' gender composition, age diversity, average age and overall experience.

## Declarations

### Author contribution statement

Bambang Tjahjadi: Conceived and designed the experiments; Wrote the paper.

Noorlailie Soewarno: Analyzed and interpreted the data; Wrote the paper.

Febriani Mustikaningtiyas: Performed the experiments; Contributed reagents, materials, analysis tools or data; Wrote the paper.

### Funding statement

This research did not receive any specific grant from funding agencies in the public, commercial, or not-for-profit sectors.

### Data availability statement

Data included in article/supplementary material/referenced in article.

### Declaration of interest statement

The authors declare no conflict of interest.

### Additional information

No additional information is available for this paper.
